# Perception of Gluten-Free Bread as Influenced by Information and Health and Taste Attitudes of Millennials

**DOI:** 10.3390/foods11040491

**Published:** 2022-02-09

**Authors:** Nomzamo Magano, Gerrie du Rand, Henriette de Kock

**Affiliations:** Department of Consumer and Food Sciences, University of Pretoria, Private Bag X20, Hatfield, Pretoria 0028, South Africa; gerrie.durand@up.ac.za (G.d.R.); riette.dekock@up.ac.za (H.d.K.)

**Keywords:** health and taste attitudes (HTAs), gluten-free (GF), bread, millennials, sensory properties, acceptability, general perception

## Abstract

Information on what drives consumers to like or dislike bread is needed to provide insight on developing gluten-free (GF) bread, using indigenous and sustainable crops in Africa, such as sorghum and millet. Consumer attitudes toward the health and taste aspects of food are major drivers of food choices. The objectives of this work were (1) to determine the health and taste attitudes (HTAs) and general perceptions of a group of millennial consumers in South Africa (*n* = 354), concerning GF breads; and (2) to determine whether HTAs affect the acceptability of sensory properties of commercial GF breads, as assessed by consumers (*n* = 173), under informed and uninformed conditions. Mean scores of the taste factors were higher compared to health factors, indicating a greater taste orientation. The sensory properties of standard wheat breads were preferred over two commercial GF breads, irrespective of the health/taste interests of consumers, or if they were informed/uninformed about the nature of the bread (GF or wheat). Knowledge that bread samples were GF reduced only the acceptability of the aroma of GF bread. GF bread was perceived as healthier, but less tasty. For this group of millennials, the sensory properties of bread was the main driver of choice.

## 1. Introduction

Health and taste are two important factors affecting food choices [1]. Taste refers to the hedonic benefit perceived, based on the sensory properties when choosing and consuming a product. In effort to measure the relative extent and importance of these two constructs (health and taste) for consumers, when it comes to food choices, Roininen, Lähteenmaäki and Tuorila [1] developed a “Health and Taste Attitude Scale” (HTAS). This questionnaire has been used worldwide to assess the health and taste attitudes of consumers in countries such as Taiwan [2], Serbia [3], Italy [4], Finland, The Netherlands, and Britain [5], but not yet in Africa. Here, the questionnaire was employed to determine the HTAs of a group of millennial consumers in South Africa (SA).

The millennial generation, or “Generation Y”, consists of people who were born between 1980 and 2000 [6]. This generation is much larger than the preceding “Generation X” [7], who account for 35.5% of the SA population [8]. Millennials are a significant market, which justifies them as the consumer group used for the study.

At the time of the study, there were two main commercial gluten-free (GF) bread brands in SA. The standard wheat sample used was one of the most consumed brands. Information on the acceptability of the sensory properties of commercial GF bread could provide insight into the development of bread products made from indigenous, GF, and climate-resilient crops, e.g., sorghum and the millets, thereby reducing the reliance on wheat imports. At the same time, it is also known that drivers of “liking” go beyond the sensory properties (“taste”) of food products [9,10,11]. Gluten-free products are perceived to be relatively healthy [12], expensive [13], firm, dry, and crumbly in texture [14,15,16]. Other factors, such as a consumer’s state of wellness, past experiences with the product, and inherent attitude towards it, are also important to consider. 

The first objective was to determine the orientation of a group of millennial consumers in South Africa towards the health and taste characteristics of food, measured using the HTAS questionnaire [1]. The second objective was to determine the consumers’ acceptance of the sensory properties of GF bread ((blind-coded; uninformed) (labeled; informed)) as influenced by the HTAs. How consumers perceived the characteristics of GF bread in general was also tested through a check-all-that-apply analysis. This information could help determine whether consumers will be driven by health or taste considerations when it comes to food choices pertaining to GF bread. It will also determine whether consumers need more education about the meaning of GF and its implications for food and consumer health.

The research covering the two objectives are presented as two phases.

This study was approved by the Ethics Committee of the Faculty of Natural and Agricultural Sciences, University of Pretoria (reference number: NAS187/2019).

## 2. Phase 1: Health and Taste Attitudes of a Group of Millennial Consumers

### 2.1. Materials and Methods 

#### 2.1.1. Participants

The participants (born between 1980 and 2000) registered on a database, where persons interested in food evaluation studies voluntarily signed up to. An invitation link with information about the study was emailed to the group. By clicking on the link, they consented to participate and were led to a questionnaire to complete via Compusense Cloud.

#### 2.1.2. Procedure

The HTAs were measured using the HTAS questionnaire [1]. The health scale consists of three factors: general health interest (eight items), light product interest (six items), and natural product interest (six items). The taste scale consists of three factors: craving for sweet foods (six items), using food as a reward (six items), and pleasure (six items). Half of the statements in each of the factors were negatively worded; the scores for these statements were hence reverse-coded prior to analysis [1]. All items were presented in English, with no changes from the original paper.

The participants rated all items on a seven-point Likert scale, ranging from (1) strongly disagree to (7) strongly agree. The items from all six factors were presented in a random order to minimise order effects, thereby increasing the validity of the results. At the end of the questionnaire, respondents were asked to indicate their gender, highest education level (grade 12, higher certificate, diploma, bachelor’s degree, or postgraduate degree), whether they had heard of gluten or not, which bread type (white, brown or seeded) they consumed the most, and whether they had an intolerance to gluten or not. They were asked to give an awareness response (true or false) to the statement “gluten-free breads are healthier than gluten-containing breads”—as was also asked in a study on the perceptions of the GF concept, by Dunn, House [12]. Respondents who indicated that they did not eat bread, had an intolerance to gluten, or had a wheat allergy (*n* = 71) were not invited for a follow-up in-sensory laboratory evaluation of bread samples.

#### 2.1.3. Statistical Analysis

The mean scores for each factor from each respondent were determined. The distribution of respondents who believed that GF bread was healthier than gluten-containing bread and those who did not was compared using binomial distribution. The factor structure of the HTA factors was determined through exploratory factor analysis using Varimax (orthogonal) with the Kaiser normalization rotation method. This method was chosen over a non-orthogonal method, because none of the component correlation matrix values exceeded |0.32| [17]. Moreover, the rotated component matrix was expressed, with the absolute factor values below 0.3 excluded [18]. Extraction was based on Eigen values greater than 1 [19]. The internal consistency (reliability) of the HTA factors was determined by calculating the coefficient alpha [20] of each factor. The statistical analyses were conducted using SPSS version 25 (IBM Corporation ^®^, New York, NY, USA).

### 2.2. Results

The HTAS questionnaire was completed online and independently by 354 millennials. Most of the respondents were female (66.6%) and a majority (56.1%) thought that GF bread was healthier than gluten-containing bread (*p* < 0.05). The majority of respondents (68.8%) completed grade 12, 20.4% completed a post-school qualification, and 11% a postgraduate degree.

#### Reliability of the Health and Taste Attitudes Questionnaire

Table 1 shows the factor loadings of the health sub-scale factors by means of a rotated component matrix. The four negatively worded items (5, 6, 7, 8) of the general health interest factor, along with item 3, loaded together on component 1, with item 5 having the highest factor loading. Items 1, 2, and 4 of the general health interest factor loaded together on component 3. The light product interest items loaded on component 2, with item 3 having the highest factor loading. Items 1, 2, 3, and 5 of the natural product interest factor had the highest factor loadings on component 4, whereas items 4 and 6 were better fitted to component 3. The Kaiser–Meyer–Olkin measure of sampling adequacy was 0.85, which is satisfactory [21]. Bartlett’s test of sphericity was significant (*p* < 0.0001) and the four extracted components cumulatively explained 54% of the variance.

Table 2 shows the factor loadings of the taste sub-scales by means of a rotated component matrix. The craving for sweet food items loaded together on component 1, with item 3 having the highest factor loading. Items 1, 2, 3, and 4 (using food as a reward) loaded together on component 2; however, items 5 and 6 were better fitted in component 3. For the pleasure factor: item 1 loaded well on component 3, while items 3 and 6 loaded together on component 4, and items 2, 4, and 5 loaded well on component 5. The Kaiser–Meyer–Olkin measure of sampling adequacy was 0.80, which is satisfactory. Bartlett’s test of sphericity was significant (*p* < 0.0001) and the five extracted components explained 57.5% of the variance.

Table 1 and Table 2 also show the coefficient alpha values, which show the internal consistency as an indication of reliability, for the HTA factors. The general health interest factor had the highest alpha (0.83) followed by craving for sweet foods (0.78). Pleasure (α = 0.51) increased to 0.54 after item 5 (“I finish my meal even when I do not like the taste of a food”) was deleted. The mean score for craving sweet foods was highest and light product interest was lowest.

## 3. Phase 2: Acceptance of Bread Samples as Influenced by Health and Taste Attitudes

### 3.1. Materials and Methods 

#### 3.1.1. Participants 

**Figure 1:**
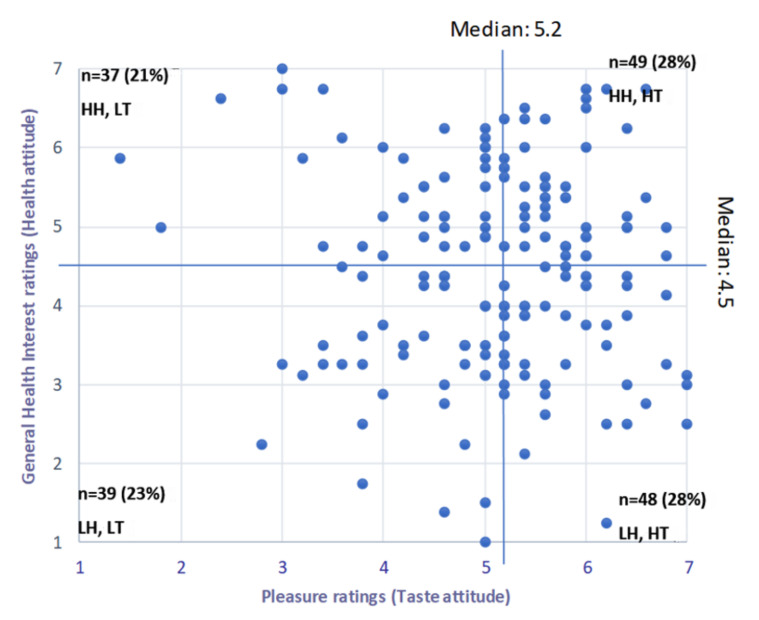
Distribution and classification of respondents based on their “general health interest” and “pleasure” attitude scores (*n* = 173) as determined by the Health and Taste Attitude Scale. Where HH, LT = high health, low taste; HH, HT = high health, high taste; LH, LT = low health, low taste and LH, HT = low health, high taste.

A subset of 173 participants were recruited from a group of 354 people from phase 1. These were people who (1) completed the HTAS questionnaire; (2) qualified to be invited to the sensory evaluation of bread, based on not having any wheat or gluten intolerances; and (3) voluntarily scheduled themselves for a sensory evaluation session, and attended. Each participant was classified into one of four HTA groups according their health (general health interest) and taste (pleasure, with item 5 deleted) attitudes (Figure 1). Thus, only one factor from the health and one factor from the taste sub-scales was considered for the classification.

#### 3.1.2. Products

Three commercial brown bread products (Supplementary Table S1) were evaluated, two were GF (GFA and GFB), and the third was a standard brown wheat (*Triticum aestivum*) bread. The flours that were used in GFA were tapioca, sorghum, and rice flours, psyllium husk, and potato and maize starches. GFB contained maize starch, golden linseed flour, legume flour, vegetable flour, whole flaxseed meal, millet flour, buckwheat flour, rice flour, and potato starch. The prices per kg of each product, at the time of purchase, was ZAR 17.13 (standard wheat), ZAR 171.72 (GFA), and ZAR 138.64 (GFB). Brown bread was the most consumed bread type as indicated by the respondents in phase 1. The bread samples were presented as round (diameter of 59 mm) portions from the crumb area of a bread slice to ensure comparability as the three bread loaves differed in size. The size differences of the slices can be seen in [22]. Samples were presented in transparent 120 mm × 180 mm Ziploc bags.

#### 3.1.3. Procedure

The evaluation was conducted in a 16-booth sensory laboratory over three consecutive days (1–3 October 2019). Samples were presented under white daylight conditions at room temperature (±21 °C). Filtered water was provided for palate cleansing. On each day, four 1-h sessions (08:30 a.m. to 12:30 p.m.) were held, and each participant received a store-voucher worth ZAR 25 afterwards as a token of appreciation. Each participant had to sign up to one time slot. Compusense Cloud (Compusense Inc., Guelph, ON, Canada) software was used to produce the sample codes, presentation orders, and to collect the ratings and comments for each sample from each participant.

The evaluation consisted of three sections (*n* = 173): 

Section 1.

Using a five-point hedonic scale (1 = dislike very much, 2 = dislike a little, 3 = not sure, 4 = like a little and 5 = like very much), the participants rated the appearance, aroma, flavour, and texture (in-mouth) attributes [23] of the bread samples, blind-coded with 3-digit random numbers. The instructions were phrased as follows: please look at/smell/taste/eat sample 123, how much do you like or dislike the appearance/smell/taste/texture of sample 123? The sample serving positions varied from panelist to panelist, according to William’s Latin square design. A rating of 1 to 3 connected the participants to a comment section where they were asked to indicate the reason for disliking the particular sample for the evaluated attribute. Ratings from 4 to 5 connected the participants to a comment section where they were asked to indicate the reason for liking the sample for the attribute in question. This section was uninformed, as the participants had no information on the nature (GF or wheat bread) of the bread samples that they were evaluating. 

Section 2.

Next, new samples of the breads (GFA, GFB, and wheat) labeled with three-digit random numbers (different to those from the first section), as well as labels identifying them as either “gluten-free bread” or “wheat bread”, were presented. The samples were all served at the same time, with sample positions varying from person to person according to William’s Latin square design. The participants were again requested to rate the appearance, aroma, flavour, and texture of the bread samples using the same five-point hedonic scale. The participants were also given the option to give overall/general comments on each of the samples after evaluating them. 

Section 3.

Participants were tasked to associate characteristics that typically apply to GF bread and wheat bread from a list of 24 options, provided in a check-all-that-apply (CATA) manner. The list of CATA words was generated based on comments obtained from a pilot sensory evaluation of the bread samples with 24 millennials (not participating in other parts of this study), and on suggestions by the researchers. 

#### 3.1.4. Statistical Analyses

SPSS version 25 (IBM Corporation^®^, New York, USA) was used for the statistical analysis of the HTAs of the respondents. The mean scores of the two factors chosen to represent health and taste orientation (general health interest and pleasure) from each respondent were determined. The median (50th percentile) score of each factor was used as the cut-off point between high and low health/taste attitude [24], creating four groups (HT, HH; HT, LH; LT, HH, and LT, LH; Figure 1). The distribution between those who believed GF bread was healthier versus those who did not was compared using binomial distribution. 

The main and interaction effects of the HTA groups, samples, and information (knowing the GF/wheat status of the samples) variables on the hedonic ratings of the sensory properties of the bread were determined through a type Ⅲ, three-way analysis of variance (ANOVA). Fisher’s least significant difference (LSD) test at *p* < 0.05 was used to compare means. Comments on the bread samples separated by HTA groups were refined using a coding method [25] and presented as word clouds, using www.wordclouds.com (Supplementary Figures S1–S6). The perceptions of participants about GF and wheat bread (association test by CATA) were analyzed using Cochran’s Q test and compared at *p* < 0.05. The ANOVA and CATA analyses were done using XLSTAT software (version 2020.1.3, Addinsoft, Long Island, NY, USA).

### 3.2. Results

Regarding the distribution of respondents, based on their health and taste attitudes—of the 354 participants who completed the HTAS questionnaire (phase 1), 283 qualified to be invited to the sensory evaluation, based on exclusion criteria defined above. Of these, 173 attended. The results presented and discussed are from the subset of 173 participants (71.1% female). Based on its relevance, only the general health interest factor was considered for classifying the participants as having a high or low health attitude. The corrected pleasure factor (item 5 not included) was used to classify participants according to either high (56% of respondents) or low (44%) taste attitude (Figure 1). The high health, low taste group had the lowest number of respondents (21%) and the two high taste groups the most (28%). More respondents (55.5%, *p* < 0.05) also thought that GF bread was healthier than gluten-containing bread.

#### 3.2.1. Liking of Sensory Properties of Bread Samples

Table 3 shows the ANOVA table of the main and interaction effects for the hedonic ratings of the sensory properties of the bread samples. The sample effect was significant for all attributes and the effect of information was only significant for hedonic ratings of the aroma. A significant interaction effect of HTA group X sample was observed for hedonic ratings of bread appearance (*p* = 0.05) and flavour (*p* = 0.04). There was no other significant interaction or main effects. The sensory attributes of the standard bread were significantly more acceptable than those of the GF breads (Table 4). When respondents knew that the bread was GF, they liked the aroma of the samples significantly less. Information did not have a significant effect on the liking of the aroma of the standard bread.

#### 3.2.2. Effect of Health and Taste Attitudes on Ratings of Bread

Table 5 shows the mean hedonic ratings for the sensory properties of the GF breads as affected by the interaction of HTA groups and samples. Hedonic ratings of the appearance of the bread (standard, GFA, and GFB) did not differ significantly for consumers in the HTHH (high taste, high health) and LTLH (low taste, low health) groups. Whereas, consumers in high taste, low health (HTLH) and low taste, high health (LTHH) groups rated the liking of the appearance of the GF breads significantly lower than the wheat bread. Liking for the flavour of GF bread was rated lower than wheat bread by all the HTA groups. The interaction between HTA groups and samples was significant for the appearance and flavour ratings of the bread samples. 

#### 3.2.3. Reasons for Liking and Disliking the Sensory Properties of the Wheat and Gluten-Free Bread Samples 

The most frequent reasons for disliking GFA were that it was dry, stale, sponge-like, rough, and hard. Those who liked the sensory properties of GFA made comments, such as healthy, sweet, spongy, and different (Supplementary Figures S1–S6). The most frequent reasons for disliking GFB were that it was dry, stale, hard, sandy, not fresh, unexpected, crumbly, bland, weird, and had an off-smell. Those who liked the sensory properties of GFB made comments such as healthy, grainy, light, fresh, different, and seeded. Reasons stated for disliking the standard bread included bland, chewy, normal/familiar. However, the terms normal/familiar, along with soft, were also frequently given as reasons for liking the standard bread.

#### 3.2.4. Perceived Characteristics of Bread Samples

Table 6 shows the comparison of the characteristics associated with GF and wheat bread as selected by participants from a CATA list, sorted in descending order of frequency of checking for GF bread. Participants thought that GF bread, compared to wheat bread, was healthy, for people with allergies, contributed to weight loss, had a foreign taste, was a source of fiber, and natural. Wheat bread, compared to GF bread, was more often cited as easy to find, affordable, a source of fiber, and tasty/delicious. 

## 4. Discussion 

The sensory properties of the standard wheat bread were preferred to GF bread; this was irrespective of the health or taste interests of consumers, and when consumers were informed or not about the GF/wheat containing “nature” of the bread. This indicates a greater taste orientation. Knowledge that bread samples were GF reduced only the acceptability of the aroma of GF bread. 

### 4.1. Hedonic Perception of Bread as Influenced by Information

Recognition of the more familiar sensory properties of the wheat bread (when identified—informed) may have reduced any phobia, leading to higher liking. Studies have shown that the sensory properties of familiar grain products (GF sorghum-based cakes) are more preferred than unfamiliar alternatives [26,27]. The GF label reduced acceptability of the aroma of the GF samples. Smelling an aroma prepares one’s body for ingestion, it is also important in the anticipation phase of eating [28,29]. The less familiar aromas of the GF samples may have triggered neophobic reactions, or they were less desirable to the consumers, considering that they could anticipate the flavour based on the aroma. Information about the nature of the bread did not impact the hedonic rating of the taste and texture. It was expected that information would increase the liking of GF bread because of the health perception associated with it. This contradictory finding supports the notion that many consumers consume food more for taste rather than for its perceived health benefits [24].

Participants preferred the aroma of GFA compared to GFB. The aroma of the GFA bread was described by a descriptive panel as toasted and sweet coffee (results not presented). These may have been the drivers of liking. Participants preferred the texture of GFB to that of GFA. The elasticity of the GFB bread texture was more similar to the wheat bread according to the descriptive sensory and texture analyses (results not presented).

### 4.2. Hedonic Rating of Bread as Influenced by HTA

Information about the nature of the bread (GF or wheat) affected the hedonic ratings of participants with a high health and high taste attitude (HTHH) differently compared to the other groups. Their likings of the aroma of the GF samples was negatively affected by information. GFB, in particular, was frequently described as having an “off-smell” by this group. This means that despite their high health attitudes, their high taste attitude reduced tolerance of the unfamiliar aroma of the GFB bread. GFB was described as having a yeasty aroma and nutty flavour by a trained sensory panel (results not presented). The HTHH group also liked the flavour of GFA more than the other groups. The flavour of the GFA bread was described to have a toasted and “sweet coffee” flavour by a trained sensory panel (results not presented). Bernstein and Rose [23], in a study on the preference of commercial whole wheat breads, found that a large number of consumers (from Lincoln, USA) liked breads that were sweet. 

Participants with high taste and low health attitudes (HTLHs) rated the flavour of the wheat bread higher compared to the other groups. The words “normal” and “soft” were the most frequent reasons for liking. Familiarity seemed to be important for this group. Consumers tended to dislike products they were not familiar with [30]. This group was also negatively influenced by information when it came to the liking of the aroma of GFB. GFB was the least liked sample in terms of aroma and flavour by all groups in both informed and uninformed conditions. This HTLH group might have preferred the more familiar wheat bread, considering that taste was important to them. They may also have associated GF bread with having less desirable sensory properties, and they were probably unwilling to compromise taste when the presumed “healthy” GF alternatives were presented [24].

Although most of the comments for GFB from all groups were negative, some participants from the low taste, high health (LTHH) group described GFB as “nice”, “regular”, and “impressive.” Moreover, participants frequently described GFB as being “healthy”. This may imply that these participants are indeed more concerned with health aspects. However, this group still showed a higher liking for the sensory properties of wheat bread compared to both GF samples. This may be because drivers of liking are mostly defined by the sensory properties of a product, followed by the health aspects [26]. This group may however be more willing to compromise the familiar and more desirable sensory properties of wheat bread for GF bread compared to other groups. 

Frequent comments on GFB by the low taste, low health (LTLH) group were “appealing”, “easy to chew”, and “normal.” These comments were rather positive compared to other groups. This may indicate that some participants from this group did not care about the taste of the GF bread as their low taste orientation suggests.

### 4.3. Perceived Characteristics of Bread Samples

The participants were of the view that GF bread was more of a specialty bread, meant for people with allergies, for weight loss purposes, and for enhancing one’s health. This coincides with Dunn, House [12], who found that students at the University of Florida (USA) had unfounded perceptions about the health benefits and the potential positive impact a GF diet had on one’s health. Instead, several studies have described the GF diet to be low in protein, rich in carbohydrates, high in fiber, and of a high glycemic index, in relation to the recommended dietary allowance for adults [31,32,33]. As such, most commercial gluten-free products are nutritionally inferior to their gluten containing alternatives.

Participants were also under the impression that GF bread is natural, organic, and less processed compared to wheat bread. It shows that the participants seemed to believe that gluten is an unnatural or a manmade ingredient added to bread [34]. These associations are not valid, but demonstrate popular misconceptions often driven by misinformed celebrities and the media in general [35]. The participants justifiably viewed wheat bread as being more palatable and accessible to them. In fact, GF bread was found to be 700 to 900% more expensive than wheat bread, explaining the lower familiarity and market share. Furthermore, it is quite challenging to replicate the desirable sensory properties (e.g., soft texture with bland flavour) of gluten-containing wheat bread using GF cereals, despite the various alternative cereal flours used. Participants also showed ignorance about the origins of the crops used to produce GF and wheat bread. Of the few participants who selected the “made with flour from sustainable crops” option, most of them associated this statement with wheat bread. In the current context of South Africa, wheat production is not sustainable, as most of it needs to be imported. The harsh climate of many parts of South Africa, coupled with the relatively poor agricultural infrastructure and high dependence on rain for crop growing, is unconducive to high yield wheat cultivation [36,37]. Therefore, the wheat supply is heavily supplemented by the global market [38]. The results show a clear need for more consumer education about gluten, its purpose in bread, potential human health effects, disadvantages and advantages of gluten-free products, and sustainable and indigenous sources of ingredients, as highlighted by Vázquez-Araújo, Chambers IV [26].

### 4.4. The Health and Taste Attitude Scale Performance in This Study

The mean scores of the taste factors ranged from 4.98 to 5.22, which were higher than the ranges for the health scale factors (4.13 to 4.52) and higher than the taste factors’ mean score ranges from studies in Taiwan (4.06 to 4.42) [4], Finland (3.4 to 4.6), the UK (4.1 to 5.0), and the Netherlands (4.0 to 5.1) [5]. However, the mean scores for health were lower than those of the studies in these countries. This may be an indication that, for this group of consumers, taste aspects are more dominant than health aspects when it comes to food. Taste was reported to be one of the most important factors consumers consider when it comes to the food they consume [1,39,40]. Light product interest scored the lowest of all factors. A similar observation was made by [5] when studying consumers in the Netherlands. The light product interest mean was 3.9 ± 1.1 in the Netherlands and here 4.1 ± 1.2. Light product interest reflects the extent to which the respondent values eating “light” foods (reduced-fat foods) for maintaining good health and physical appearance [1]. Most of the participants in this study did not necessarily view consuming light products (as evaluated by this particular scale for measuring light product interest) as particularly important for their health.

Exploratory factor analysis revealed that the light product interest factor was stable as all of its items loaded well on component 2. A similar pattern was found by [4] in a study of the health attitudes of 1224 Italian consumers. However, unlike in the studies by [3,4], the general health interest factor of this study had items with high loadings in two separate components. General health interest therefore formed two constructs, one had all of the negative (R) items (items 5, 6, 7, 8 along with item 3), which implied little or no care for one’s health, except item 3, which also showed the lowest communality in the entire questionnaire. The other construct contained items 1, 2, and 4, which alluded to one being meticulous about the healthfulness of a diet. Item 5 of the general health interest factor (“I eat what I like, and I do not worry about healthiness of food”) had the highest factor loading and this may mean that this item best represented the general health interest of the participants in this research. For natural product interest, items 4 and 6 which refer to processed foods and foods containing additives, had high loadings on component 3—thereby forming a separate construct specific to an interest in whether food was processed or contained additives or not. Similar results were noted by [5] in their study of the health attitudes of Finnish (*n* = 467), British (*n* = 361), and Dutch (*n* = 477) consumers. 

For taste factors, craving for sweet foods was stable in that all items loaded strongly on component 1. The same stability was also noted by [3] in Serbia and [5] in Finland. However, in the research done by [41] and [4], craving for sweet foods formed two separate constructs, one based on items related to personal craving and the other relating to other people’s cravings. Similar to a study with consumers in Great Britain [5], items 1, 2, 3, and 4 of the ‘using food as a reward’ factor in this study loaded well on component 2 but items 5 and 6 were better fitted on component 3. These two items are related to emotional eating (when emotional states and situations affect food intake) [42]. The pleasure factor however, yielded three constructs—this may also be the reason for its relatively low reliability. Item 1 of pleasure loaded well on component 3 with the two emotional eating items from the using food as a reward factor. This item addresses one’s standpoint on whether food should be a source of pleasure or not, which may have also been interpreted as a form of emotional eating by the participants. Items 3 and 6 of the pleasure factor loaded well on component 4. These items refer to eating delicious foods specifically on weekends. Items 2, 4, and 5 loaded well on component 5, these items deal with the sensory appeal or sensory benefits perceived from food. Item 5 had the lowest coefficient alpha. This is an indication that item 5 may have represented something other than pleasure. Responses to item 5 (“I finish my meal even when I do not like the taste of food”) may have been affected by other inherent values, such as food waste consciousness or acknowledging the economic benefit of consuming all food served; and not necessarily the motivation to obtain pleasure from food. Item 5 also loaded poorly with lowest communality for consumers in Great Britain [5]. 

The HTA factors had acceptable coefficient alpha values, although rather weak for the pleasure factor (0.51). The coefficient alpha determines the internal consistency of the items of the scale and the closer the value is to one, the more reliable the scale is [43]. George and Mallery [44] suggested that a scale with coefficient alpha value <0.50 is unacceptable. Similar to [2], it was found that after deleting item 5 of the pleasure factor, the coefficient alpha increased from a borderline satisfactory 0.51 to a more acceptable 0.54. The light product interest, natural product interest, craving for sweet foods and using food as a reward factors had higher coefficient alphas than those measured in studies with consumers in the UK and the Netherlands by [5]. This means that these four factors were more reliable in measuring their respective constructs compared to the study done by [5].

The general health interest factor was used for classifying the participants (*n* = 173) into having either a high or a low health attitude and the pleasure factor was used for classifying participants according to taste attitudes. This was done based on face validity by the researchers. The factors that were left out would presumably not contribute to consumers attitudes towards GF bread. For example, natural product interest has an item referring to organically grown vegetables and light product interest has two items which deal with cholesterol, both are not related to health aspects of GF bread. Sabbe, Verbeke [24] did the same when studying health-related attitudes and beliefs of Belgian consumers connected to the acceptance of fruit juices with added acai. Cox, Melo [45] used only three of the six factors (general health interest, pleasure, and using food as a reward) to determine the effect of taste, information, and attitudes on the acceptance of Brassica vegetables amongst Australian consumers. 

### 4.5. Limitations of the Study

Firstly, the participants here were a “convenience sample” of educated millennials in urban SA who were interested in participating in food studies. The classification of consumers into the four HTA groups was based on a single tool, i.e., the HTA scales. This is limiting because there may be other health and taste factors that consumers consider (in the South African context), which may not have been part of the HTA scales. Unfortunately, the pleasure factor was here, and based on studies with other populations in different countries [4,5,39], the weakest of all HTA factors, thereby further weakening the taste category. Moreover, the unequal number of negatively worded (2) and positively worded (3) items on the pleasure scale after the deletion of item 5 (negatively worded) may have compromised response balance [46]. Furthermore, an instrument that is more specific to GF products is needed and may have been more appropriate than the HTA scale, which is more general.

The bread samples evaluated did not represent the full slice or loaf that would be purchased in store. They also represented only two of the many recipes and technological methods used in other parts of the world to produce GF brown bread. The participants only evaluated a disk-shaped portion of the crumb of the bread and not the full slice, which includes the crust. The area/size differences of the slices would have been a driver of liking or disliking in terms of appearance. The crusts were also excluded, a factor that will always be considered by consumers, in terms of appearance, aroma, flavour, and texture.

The use of a five-point scale can be a limitation as it has relatively little room for participants to express the full range of their hedonic perception [47]. However, a user-friendly test is important as it simplifies the consumer’s task of evaluating, rating, and providing comments on the product [48].

## 5. Conclusions

The study highlighted that young consumers in South Africa are much more familiar with wheat than GF bread. They also prefer the sensory properties of wheat bread regardless of their health and taste attitudes. Sustainably produced GF bread should therefore mimic wheat bread as much as possible to increase acceptance. There are many misconceptions about the benefits of consuming GF bread, demonstrating the need for education and science-based information on the topic.

The HTA subscales were reliable, i.e., had relatively high internal consistencies. However, the two subscales used for predicting did not succeed in predicting “liking” for GF bread. Factor analysis showed that the factor structure did not quite hold, which may have compromised the validity of the questionnaire. It may be useful to develop an updated HTA questionnaire that would better reflect current views and attitudes, using insights from African consumers. Moreover, analysis of the HTAs of these consumers revealed that they gravitate more towards taste than health aspects pertaining to food, which confirms the results of the acceptability of the GF bread results.

This study gives insight on aspects, which could be used to market GF bread made from climate-smart crops. These include highlighting the economic sustainability of the crops from which the flour is derived and the fact that the products are made from indigenous and climate smart crops, instead of merely stating that they are GF. Information on the drivers of liking or disliking of commercial GF bread obtained from this study could inform the development of bread products made from GF climate-smart crops that are indigenous to Africa. Research and development to improve the sensory properties and economic manufacture of GF bread from locally grown (maize) and/or indigenous crop flours (sorghum, millets, cowpeas) are critically needed for sustainability and to improve food security.

## Figures and Tables

**Table 1:** Exploratory factor analysis of the health attitudes of a group of millennial consumers (*n* = 354) in South Africa.

		$\bar{x}$ ± σ	α	F1	F2	F3	F4	h^2^
**General Health Interest**		4.5 ± 1.2	0.83					
1	I am very particular about the healthiness of food			0.53	0.05	**0.55**	0.1	0.6
2	I always follow a healthy and balanced diet			0.38	−0.09	**0.68**	−0.06	0.62
3	It is important for me that my diet is low in fat			**0.48**	0.31	0.16	0.04	0.36
4	It is important for me that my daily diet contains a lot of vitamins and minerals			0.34	0.04	**0.62**	0	0.5
5R	I eat what I like, and I do not worry much about the healthiness of food			**0.73**	0	0.26	0.12	0.62
6R	I do not avoid foods, even if they may raise my cholesterol			**0.68**	0.03	0.03	0.27	0.54
7R	The healthiness of food has little impact on my food choices			**0.7**	0.04	0.2	0.07	0.54
8R	The healthiness of snacks makes no difference to me			**0.71**	0	0.17	0.03	0.54
**Light Product Interest**		4.1 ± 1.1	0.77					
1R	In my opinion, the use of light products does not improve one’s health			0.14	**0.62**	−0.1	0.18	0.45
2R	I do not think that light products are healthier that conventional products			0.14	**0.66**	−0.1	0.16	0.49
3	I believe that eating light products keeps one’s cholesterol level under control			−0.06	**0.79**	0.06	0.1	0.64
4R	In my opinion, light products don’t help to drop cholesterol levels			0.01	**0.68**	−0.03	0.18	0.5
5	I believe that eating light products keeps one’s body in good shape			0.09	**0.72**	0.02	0	0.53
6	In my opinion, by eating light products, one can eat more without getting too many calories			−0.14	**0.57**	0.22	−0.19	0.43
**Natural Product Interest**		4.3 ± 1.2	0.74					
1R	I do not care about additives in my daily diet I try to eat foods that do not contain additives			0.35	−0.08	0.37	**0.54**	0.56
2R	In my opinion, organically grown foods are not better for my health than those grown conventionally			0.12	0.21	−0.08	**0.75**	0.62
3R	In my opinion, artificially flavoured foods are not harmful for my health			0.11	0.11	0.11	**0.75**	0.6
4	I try to eat foods that do not contain additives			0.18	0.03	**0.67**	0.39	0.64
5	I would like to eat only organically grown vegetables			0.04	0.13	0.29	**0.58**	0.44
6	I do not eat processed foods, because I do not know what they contain			0.07	0.01	**0.71**	0.19	0.55
Variance explained by each factor (%)				25.58	14.68	7.54	6.08	53.88

Negatively worded statements have an “R” after the item number. Mean ± standard deviation denoted by $\bar{x}$ ± σ. Coefficient alpha denoted by α and 1 = disagree strongly and, 7 = agree strongly. Communalities are represented by h^2^. Kaiser–Meyer–Olkin Measure of sampling adequacy = 0.85. Bartlett’s test of sphericity: *p* < 0.0001. Bold values signify the highest loading of an item in a four-factor solution.

**Table 2:** Exploratory factor analysis of the taste attitudes of a group of millennial consumers (*n* = 354) in South Africa.

		$\bar{x}$ ± σ	α	F1	F2	F3	F4	F5	h^2^
**Craving for Sweet Foods**		5.2 ± 1.3	0.78						
1R	In my opinion it is strange that some people have cravings for chocolate			**0.71**	0.03	0.23	−0.06	−0.02	0.56
2R	In my opinion it is strange that some people have cravings for sweets			**0.76**	0.08	0.11	0	−0.01	0.6
3R	In my opinion it is strange that some people have cravings for ice-cream			**0.78**	0.03	0.18	−0.11	0.1	0.66
4	I often have cravings for sweets			**0.55**	0.36	−0.28	0.29	0.01	0.61
5	I often have cravings for chocolate			**0.58**	0.42	−0.11	0.27	−0.06	0.6
6	I often have cravings for ice-cream			**0.67**	0.2	−0.09	0.1	0.1	0.52
**Using Food as a Reward**		5.0 ± 1.1	0.76						
1	I reward myself by buying something really tasty			0.11	**0.8**	0.18	0.14	−0.01	0.7
2	I indulge myself by buying something really delicious			0.15	**0.78**	0.04	0.11	0.06	0.65
3	When I am feeling down I want to treat myself with something really delicious			0.22	**0.7**	0.16	0.08	0	0.57
4R	I avoid rewarding myself with food			0.06	**0.65**	0.39	0	0.04	0.58
5R	In my opinion, comforting myself by eating is self-deception			0.11	0.07	**0.58**	0.24	−0.14	0.43
6R	I try to avoid eating delicious food when I am feeling down			0.03	0.37	**0.63**	−0.07	0.01	0.54
**Pleasure**		5.0 ± 0.9	0.51						
1R	I do not believe that food should always be a source of pleasure			0.09	0.17	**0.57**	0.19	0.16	0.42
2R	The appearance of food makes no difference to me			0.08	0.02	0.19	−0.02	**0.7**	0.53
3	It is important for me to eat delicious food on weekdays as well as weekends			0.07	−0.03	0.33	**0.75**	0.04	0.69
4	When I eat, I concentrate on enjoying the taste of food			−0.08	0.21	−0.06	0.32	**0.62**	0.54
5R	I finish my meal even when I do not like the taste of a food			0.07	−0.1	−0.11	−0.01	**0.7**	0.52
6	An essential part of my weekend is eating delicious food			−0.01	0.3	0.06	**0.73**	0.12	0.64
Percentage variance explained (%)				24.98	11.37	8.37	6.82	5.94	57.48

Negatively worded statements, “R” after the item number. Mean ± standard deviation denoted by $\bar{x}$ ± σ. Coefficient alpha denoted by α and 1 = disagree strongly and, 7 = agree strongly. Communalities are represented by h^2^. Kaiser–Meyer–Olkin measure of sampling adequacy = 0.80. Bartlett’s test of sphericity: *p* < 0.0001. Values marked bold signify the highest loading of an item in a five-factor solution.

**Table 3:** ANOVA table for the main effects and interaction effects of health and taste attitude groups, samples, and information about gluten-free/wheat bread status of bread on the sensory attributes (appearance, aroma, flavour, and texture) as evaluated by consumers (*n* = 173).

			*p*-Value			
	DF	Error DF	Appearance	Aroma	Flavour	Texture
HTA group	3	20	NS	NS	NS	NS
Sample	2	21	**0.000**	**<0.0001**	**<0.0001**	**<0.0001**
Info	1	22	NS	0	NS	NS
HTA group * Sample	6	17	**0.052**	NS	**0.037**	NS
HTA group * Info	3	20	NS	NS	NS	NS
Sample * Info	2	21	NS	NS	NS	NS
HTA group * Sample * Info	6	17	NS	NS	NS	NS

Where * = interaction and NS = not significant. The *p*-values highlighted in bold were significant at *p* < 0.05. HTA = health and taste attitude.

**Table 4:** Mean (±standard deviation) hedonic ratings for the sensory properties of the gluten-free and wheat brown bread in informed and uninformed conditions combined.

	Appearance	Aroma	Flavour	Texture
Standard wheat bread	3.9 ^a^ ± 1.1	4.0 ^a^ ± 1.0	4.1 ^a^ ± 1.1	3.9 ^a^ ± 1.3
Gluten-Free A (GFB)	3.5 ^b^ ± 1.3	3.8 ^b^ ± 1.2	3.1 ^b^ ± 1.4	2.7 ^c^ ± 1.5
Gluten-Free B (GFB)	3.6 ^b^ ± 1.3	2.8 ^c^ ± 1.4	3.0 ^b^ ± 1.4	3.2 ^b^ ± 1.4

Means in a column with different superscripts are significantly different (*p* < 0.05). Where 1 = dislike very much, 5 = like very much.

**Table 5:** Mean hedonic ratings of the bread samples as affected by health and taste attitudes (*n* = 173).

	Appearance	Aroma	Flavour	Texture
HTHH * Standard	3.8 ^abc^	3.9	3.9 ^b^	3.8
HTLH * Standard	3.9 ^ab^	3.9	4.3 ^a^	3.9
LTHH * Standard	4.1 ^a^	4.1	4.1 ^ab^	4.3
LTLH * Standard	3.8 ^abc^	4	3.9 ^b^	3.6 ^bc^
HTHH * GFA	3.7 ^abc^	3.8	3.3 ^c^	2.8
HTLH * GFA	3.3 ^d^	3.7	2.9 ^cd^	2.7
LTHH * GFA	3.6 ^bcd^	3.8	3.2 ^cd^	2.8
LTLH * GFA	3.5 ^cd^	4.1	3.1 ^cd^	2.6 ^f^
HTHH * GFB	3.6 ^bcd^	2.7	2.8 ^d^	3.1
HTLH * GFB	3.5 ^cd^	3	2.9 ^d^	3
LTHH * GFB	3.3 ^d^	2.7	2.9 ^cd^	3.2
LTLH * GFB	3.8 ^abc^	3	3.2 ^cd^	3.3
*p*-value	0.052	0.248	0.037	0.236

Mean ratings with different superscript letters (abc) significantly different, across all HTA groups and samples (*p* < 0.05). HTHH = high health, high taste; HTLH = high taste, low health; LTHH = low taste, high health and LTLH = low taste, low health. Where 1 = dislike very much, 5 = like very much and * = interaction.

**Table 6:** Comparison of the frequency of the selection of characteristics (% of consumers out of *n* = 173) associated with gluten-free and wheat bread from a check-all-that-apply (CATA) list.

Characteristics	Gluten-Free (%)	Wheat Bread (%)
Healthy *	75	32
For people with allergies *	71	4
Contributes to weight loss *	56	8
Foreign taste *	55	3
Source of fiber *	47	68
Natural *	45	28
Organic *	43	18
For people with coeliac disease *	32	2
Filling *	31	55
Bitter taste *	25	3
Appealing *	24	49
Processed *	24	52
Tasty/delicious *	23	62
Source of protein ^NS^	20	19
Made with flour from sustainable crops *	17	31
Salty ^NS^	15	13
Sweet taste *	13	36
Made with flour from indigenous crops ^NS^	13	17
Many options available *	11	57
Easy to find *	6	85
Convenient *	5	74
Affordable *	4	84
Leaves you feeling bloated *	4	31
Source of fat *	4	15

* Significant difference (*p* < 0.05) according to Cochran’s Q test. ^NS^ no significant difference (*p* > 0.05).

## Data Availability

The datasets generated for this study are available on request to the corresponding author.

## Supplementary Materials

The following are available online at https://www.mdpi.com/article/10.3390/foods11040491/s1. Table S1: Ingredients, nutritional information (per 100 g) and price (per kg) of gluten-free commercial brown bread as per the labels; Figure S1: Word cloud analyses of the reasons for disliking the sensory properties of the control brown wheat bread by consumers (*n* = 173) in the different health and taste attitudes groups when they did not have knowledge of the gluten-free/wheat bread nature of the samples; Figure S2: Word cloud analyses of the reasons for liking the sensory properties of the control brown wheat bread by consumers (*n* = 173) in the different health and taste attitudes groups when they did not have knowledge of the gluten-free/wheat bread nature of the samples; Figure S3: Word cloud analyses of the reasons for disliking the sensory properties of sample GFA gluten-free brown bread by consumers (*n* = 173) in the different health and taste attitudes groups when they did not have knowledge of the gluten-free/wheat bread nature of the samples; Figure S4: Word cloud analyses of the reasons for liking the sensory properties of sample GFA gluten-free brown bread by consumers (*n* = 173) in the different health and taste attitudes groups when they did not have knowledge of the gluten-free/wheat bread nature of the samples; Figure S5: Word cloud analyses of the reasons for disliking the sensory properties of sample GFB gluten-free brown bread by consumers (*n* = 173) in the different health and taste attitudes groups when they did not have knowledge of the gluten-free/wheat bread nature of the samples; Figure S6: Word cloud analyses of the reasons for liking the sensory properties of sample GFB gluten-free brown bread by consumers (*n* = 173) in the different health and taste attitudes groups when they did not have knowledge of the gluten-free/wheat bread nature of the samples.
