# Supplementary material for: Perception of Gluten-Free Bread as Influenced by Information and Health and Taste Attitudes of Millennials
Source: Foods. 2022 Feb 9;11(4):491. doi: 10.3390/foods11040491 (PMC8871432; doi:10.3390/foods11040491)
Supplement: Supplementary file 1 [file foods-11-00491-s001.zip › foods-1490096-supplementary.pdf]

Supplementary material

Perception of the sensory properties of gluten-free bread as influenced by the health and taste attitudes of millennial consumers

Nomzamo Magano [1,*], Gerrie du Rand [1] and Henriette de Kock [1]

[1] Department of Consumer and Food Sciences, University of Pretoria, Private Bag X20, Hatfield, Pretoria 0028, South Africa; gerrie.durand@up.ac.za; riette.dekock@up.ac.za

* Correspondence: nomzamo.magano@gmail.com

Received: 16 November 2021; Accepted: 17 December 2021; Published:

Table S1. Ingredients, nutritional information (per 100 g) and price (per kg) of gluten-free commercial brown bread as per the labels.

	Ingredients	Energy (kJ)	Protein (g)	Carbohydrate (g)	Total sugar (g)	Total fat (g)	Dietary fibre (g)	Total sodium (mg)
**Standard wheat brown bread**	Brown bread wheat* flour (gluten), water, yeast, salt, may contain acidity regulator seasonally, non-hydrogenated vegetable fat (palm fruit), soybean flour, preservative (calcium propionate), emulsifiers (vegetable origin), flavour enhancer, enzymes (non-animal origin), flour improver, mineral salts (electrolytic iron and zinc oxide) and vitamins (vitamin B3, vitamin B6, vitamin B1, vitamin B2, vitamin A and folic acid)	980	9.1	40	4.2	2.4	6.4	337
**Gluten-free brown bread A**	Tapioca flour, sorghum flour, rice flour, psyllium husk, potato starch, sugar, egg powder, maize starch, fibre, vegetable oil (sunflower seed), emulsifier (methocel), yeast, preservative (citric acid), salt	1030	4.5	41.9	1.4	4.3	10.3	542
**Gluten-free brown bread B**	Water, maize starch, golden linseed flour, gluten-free flour (contains: legume flour, vegetable flour, whole flaxseed meal, millet flour, buckwheat flour, flavouring), rice flour, potato starch, vegetable fat (palm fruit), sugar, free range egg, yeast, emulsifier, modified starch, salt, psyllium husk, cultured dextrose, preservatives: calcium propionate, potassium sorbate	1085	4.3	28	1.9	11.5	6.8	365

* Triticum aestivum

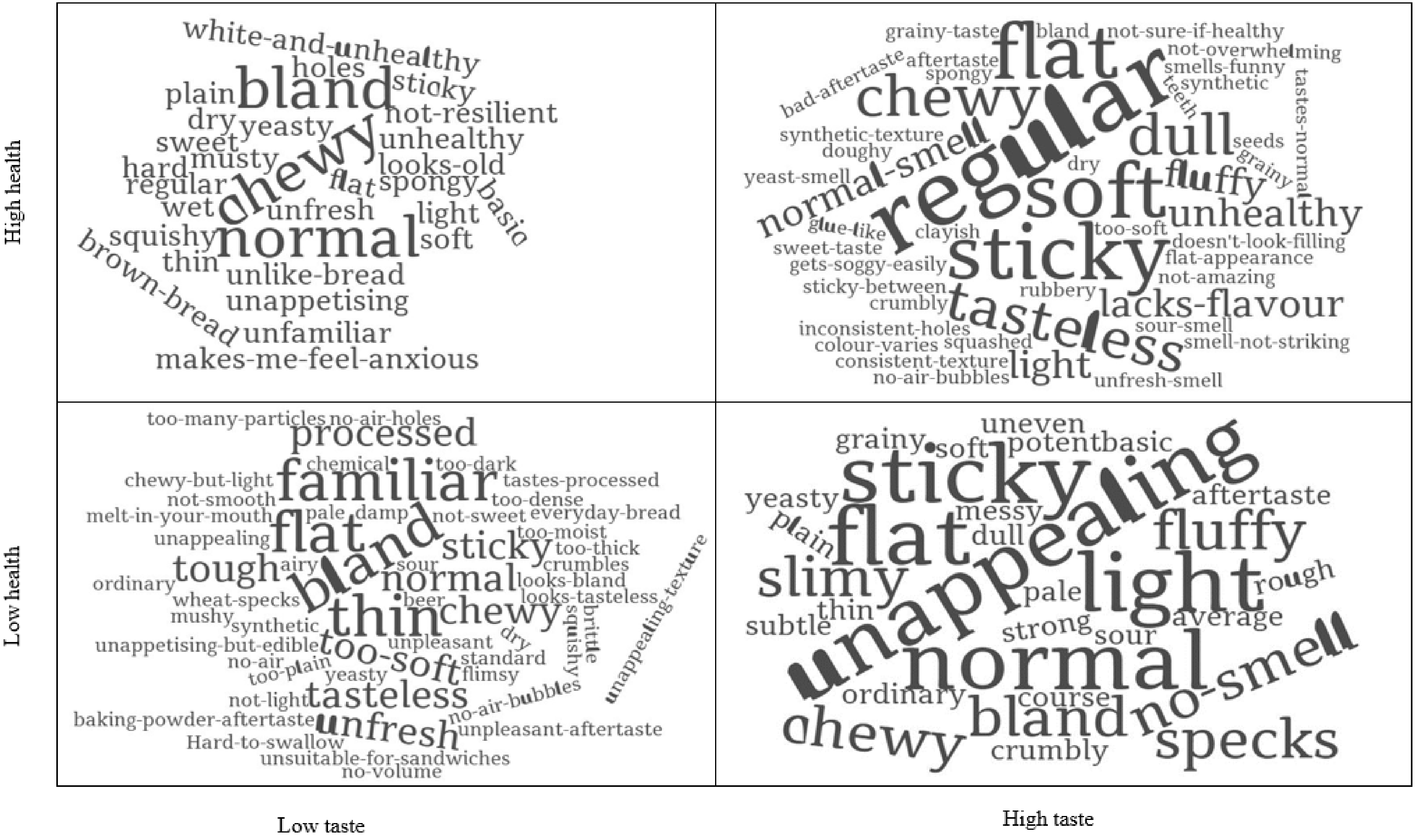

Figure S1: Word cloud analyses of the reasons for disliking the sensory properties of the control brown wheat bread by consumers (*n* =173) in the different health and taste attitudes groups when they did not have knowledge of the gluten-free/wheat bread nature of the samples. The size of the words are related to the frequency of use- the larger a word, the more frequently it was used by the consumers. *n* (LHLT) = 39; *n* (LHHT) = 48; *n* (HHLT) = 37; *n* (HHHT) = 49. Where HHHT = high health, high taste; HTLH = high taste, low health; LTHH = low taste, high health and LTLH = low taste, low health.

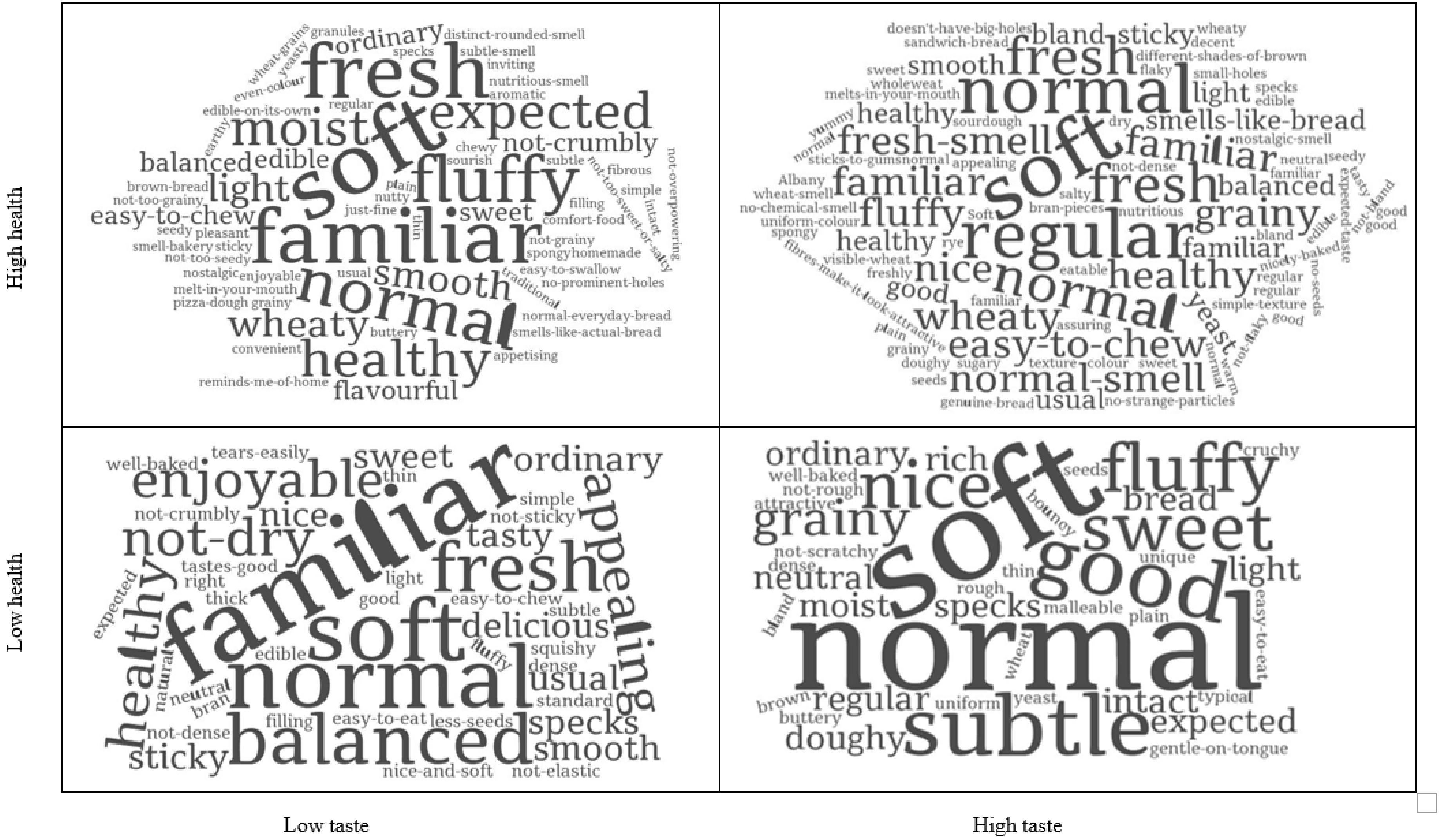

Figure S2: Word cloud analyses of the reasons for liking the sensory properties of the control brown wheat bread by consumers (n = 173) in the different health and taste attitudes groups when they did not have knowledge of the gluten-free/wheat bread nature of the samples. The size of the words are related to the frequency of use- the larger a word, the more frequently it was used by the consumers. n (LHLT) = 39; n (LHHT) = 48; n (HHLT) = 37; n (HHHT) = 49

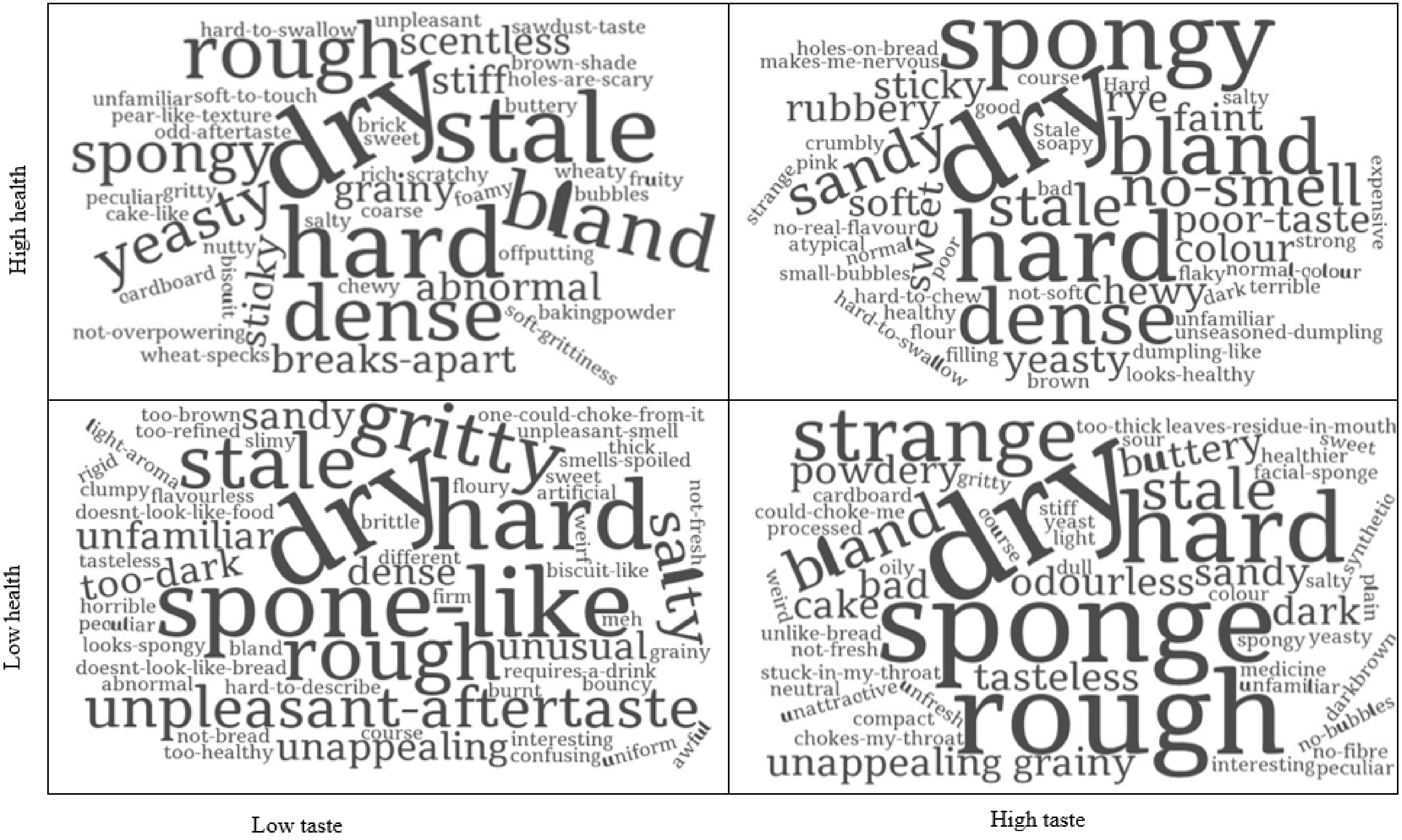

Figure S3: Word cloud analyses of the reasons for disliking the sensory properties of sample GFA gluten-free brown bread by consumers (n = 173) in the different health and taste attitudes groups when they did not have knowledge of the gluten-free/wheat bread nature of the samples. The size of the words are related to the frequency of use- the larger a word, the more frequently it was used by the consumers. n (LHLT) = 39; n (LHHT) = 48; n (HHLT) = 37; n (HHHT) = 49

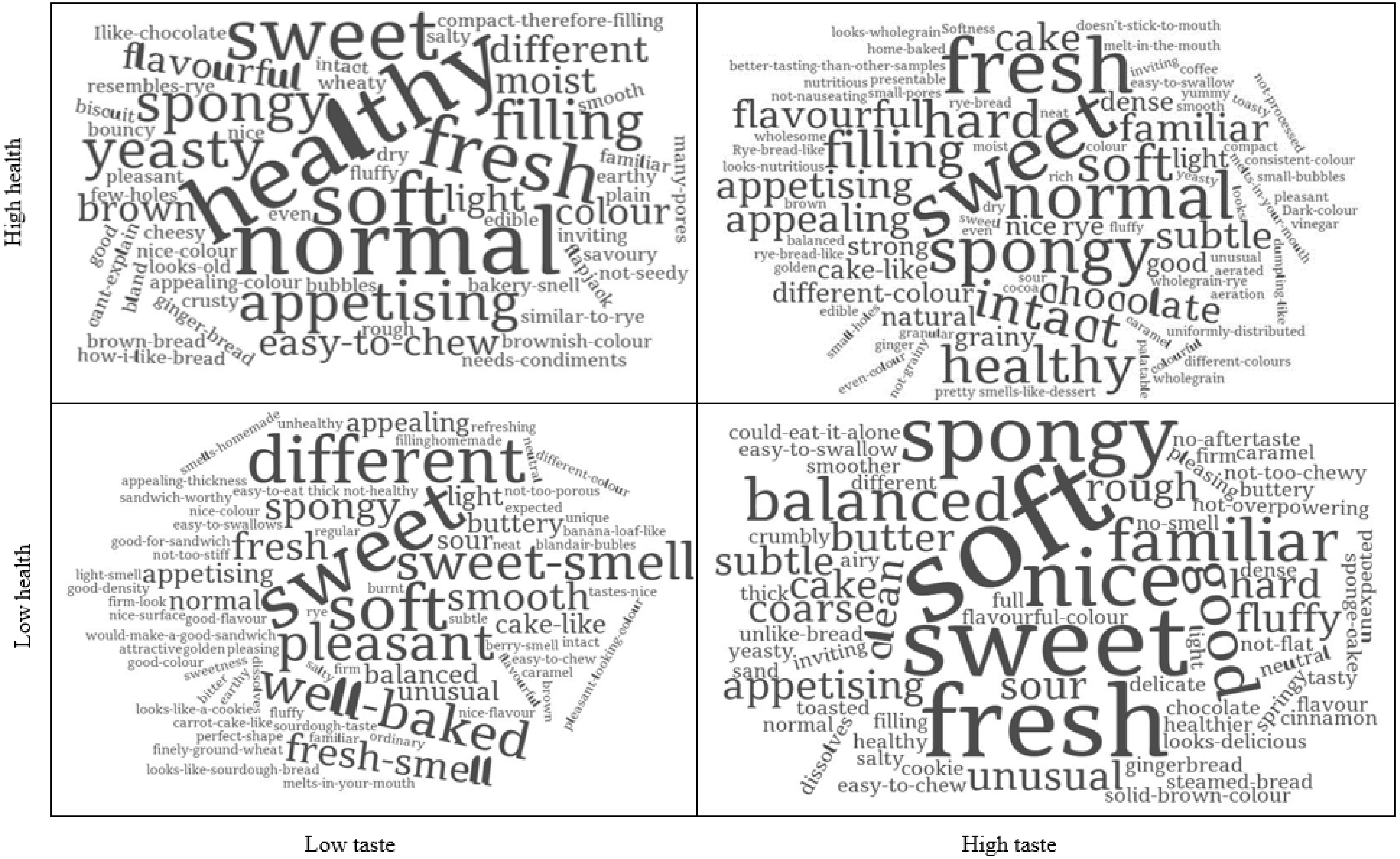

Figure S4: Word cloud analyses of the reasons for liking the sensory properties of sample GFA gluten-free brown bread by consumers (*n* = 173) in the different health and taste attitudes groups when they did not have knowledge of the gluten-free/wheat bread nature of the samples. The size of the words are related to the frequency of use- the larger a word, the more frequently it was used by the consumers. *n* (LHLT) = 39; *n* (LHHT) = 48; *n* (HHLT) = 37; *n* (HHHT) = 49

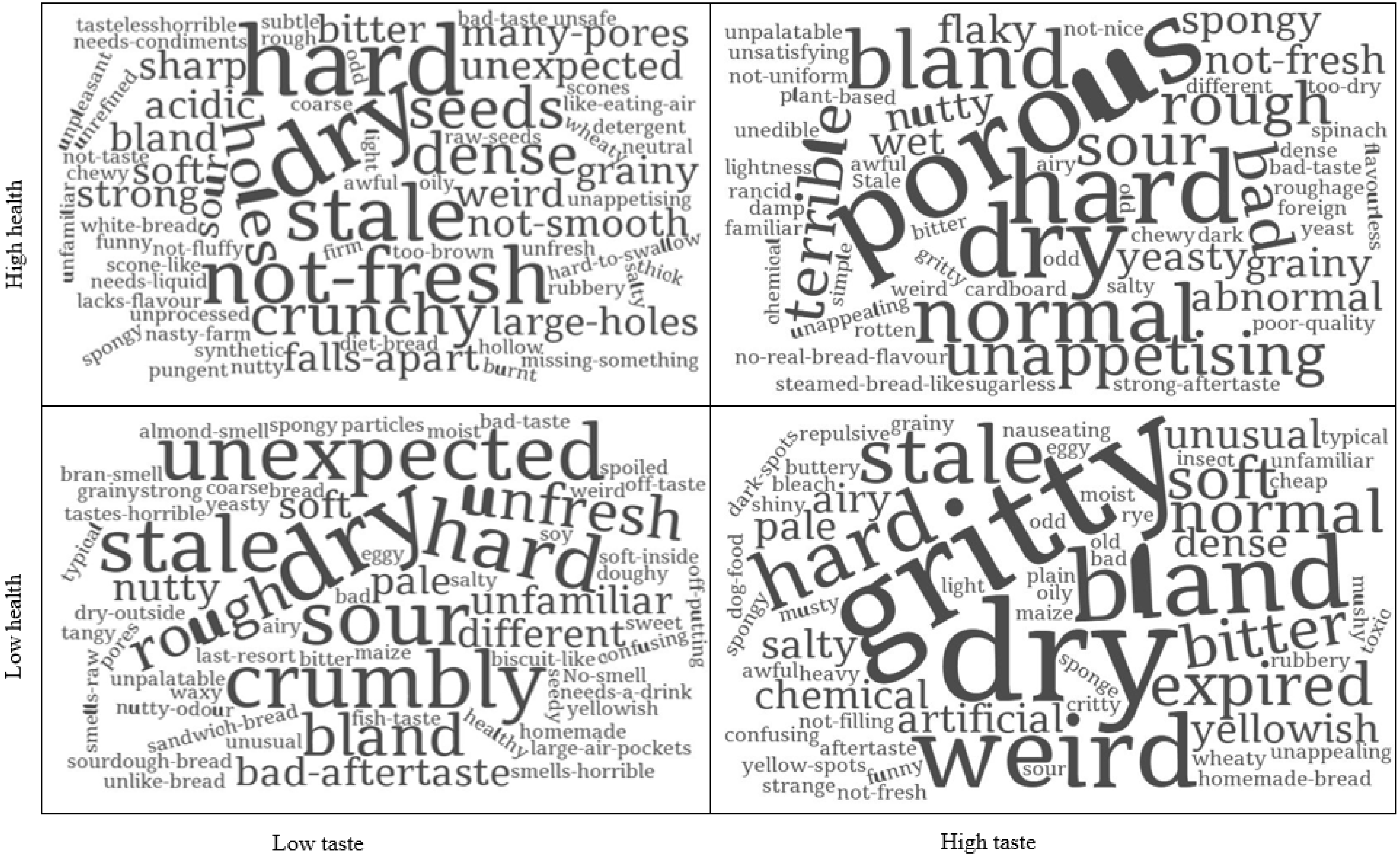

Figure S5: Word cloud analyses of the reasons for disliking the sensory properties of sample GFB gluten-free brown bread by consumers (*n* = 173) in the different health and taste attitudes groups when they did not have knowledge of the gluten-free/wheat bread nature of the samples. The size of the words are related to the frequency of use- the larger a word, the more frequently it was used by the consumers. *n* (LHLT) = 39; *n* (LHHT) = 48; *n* (HHLT) = 37; *n* (HHHT) = 49

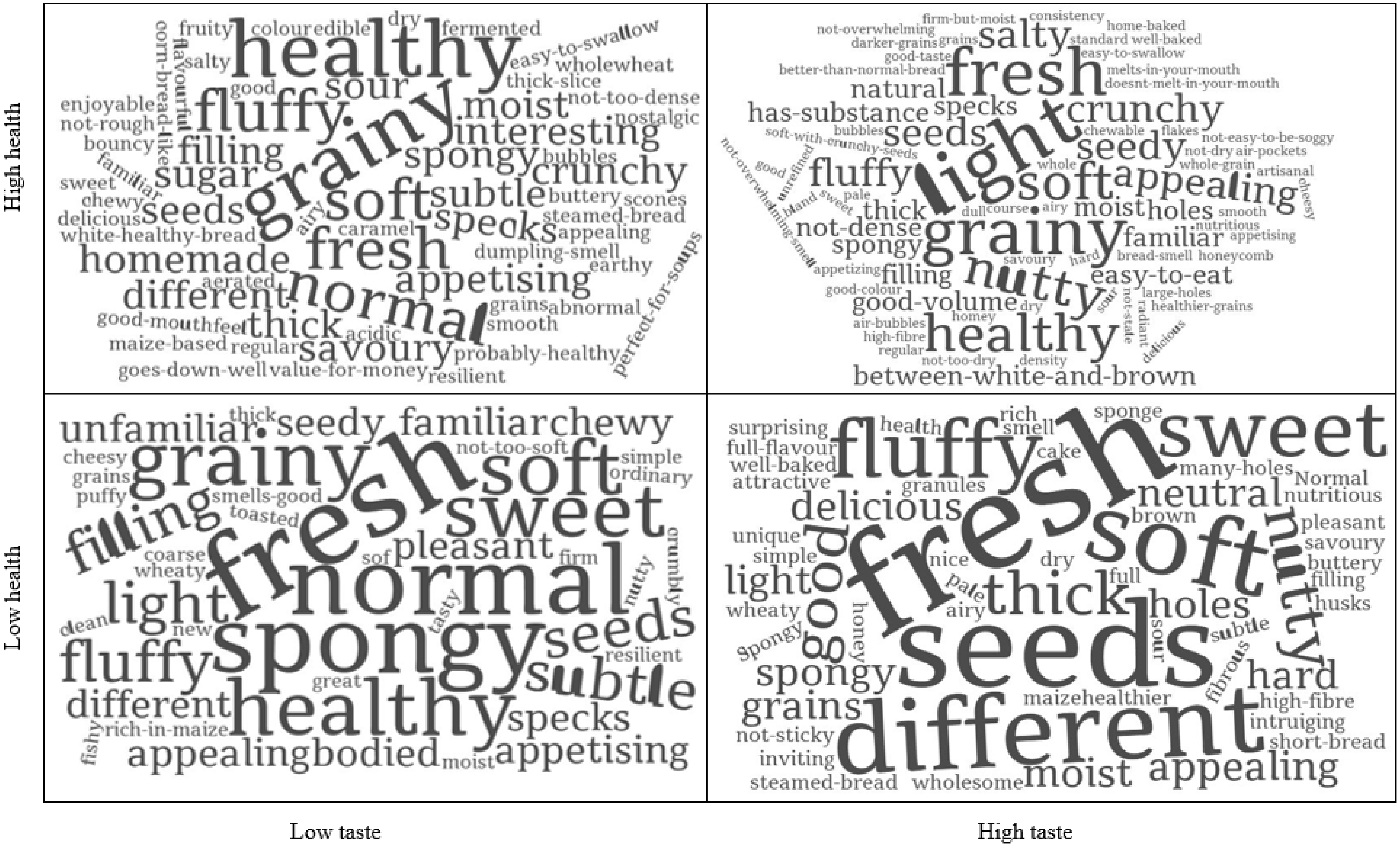

Figure S6: Word cloud analyses of the reasons for liking the sensory properties of sample GFB gluten-free brown bread by consumers (n = 173) in the different health and taste attitudes groups when they did not have knowledge of the gluten-free/wheat bread nature of the samples. The size of the words are related to the frequency of use- the larger a word, the more frequently it was used by the consumers. n (LHLT) = 39; n (LHHT) = 48; n (HHLT) = 37; n (HHHT) = 49
